# The Impact of the University Classroom on Managing the Socio-Educational Well-being: A Global Study

**DOI:** 10.3390/ijerph17030931

**Published:** 2020-02-03

**Authors:** Mariana-Daniela González-Zamar, Luis Ortiz Jiménez, Adoración Sánchez Ayala, Emilio Abad-Segura

**Affiliations:** 1 Department of Education, University of Almeria, 04120 Almeria, Spain; 2 Department of Economics and Business, University of Almeria, 04120 Almeria, Spain

**Keywords:** higher education, classroom, well-being, management, health, scientific research

## Abstract

The university learning classroom, in addition to a space for activities and architectural object, has a direct impact on the academic motivation, well-being and social relationships of the students. Thus, the link between the university classroom and the management of the socio-educational well-being of the student, in accordance with the principles of well-being theory, is a challenge that the current university must manage. The progress of worldwide research on this topic has been studied during the period 2004–2018. For this aim, a bibliometric study of 1982 articles has been applied. The results provide data of the scientific productivity of the journals, authors, institutions and countries that contribute to this research. The evidence reveals growing interest, especially in the last six years. The main category is Social Sciences. The most productive journals are *Computers and Education*, *American Journal of Pharmaceutical Education*, and *Theory into Practice*. The author with most articles is Reddy, from Rutgers University. The most productive institution is the University of Virginia. The United States is the country with most academic publications, citations and with most international collaborations in its works. Worldwide research has followed an increasing trend, with optimum publication levels in latest years.

## 1. Introduction

In recent decades, the progress reached in educational theories and paradigms, in addition to the development of information and communication technologies (ICT), have led to the need to transform the organization of learning spaces in higher education institutions (HEIs) [1,2]. This has allowed improving the physical, environmental, technological and social conditions of university classrooms [3].

Nowadays, the classroom remains the physical framework that symbolizes educational pedagogy. It is the main element on which school buildings are projected [4,5]. Therefore, the university, as a social and cultural space, must adapt to the needs of the students. This circumstance requires a leap in quality in the face of society as an architectural, environmental and sustainable paradigm. In this sense, institutions must respond to the transformation of university learning spaces and their campus [6,7,8,9].

In this context, learning spaces are not understood as a simple volumetric container of activities, but their concept goes beyond a mere architectural object. Appropriate for students and teachers, it manages to influence academic motivation, well-being and social relationships [10,11]. Since the classical definition of the university as a community of learning and teaching, knowledge has become one of the main instruments for the development of societies. For this reason, universities have been forced to answer the questions raised from the various sectors among which they exercise influence [12,13,14]. Consequently, HEIs have contributed to the development of knowledge societies or economies from different perspectives, such as politics and research, among others.

In this same order, the link of the university classroom with the management of the socio-educational well-being of the student must be established according to the principles of the well-being theory, as one of the challenges to be met by the current university. The interest in the well-being and quality of life of students focuses on fully developing their capabilities and potentials. Indicators such as happiness, health and sociability allow you to focus on a healthy and sustainable university [15,16].

The revised literature has found the terminology around the main concept of research. In this sense, the concept of the classroom refers to the basic cell that is part of learning, where individuals are related according to certain physical, human, cultural and social circumstances and situations. In the classroom, diverse and meaningful learning modes are generated. The classroom therefore does not exist without the network of relationships between students, teachers, objects and facts [17,18]. At the same time, it includes the historical, cultural and social variables that characterize and define it, and contains the relationship between educational agents and the affective bond between them all causing positive reactions in the brain.

Likewise, the term socio-educational well-being refers both to the psychological experience of happiness of the university student, due to the control and perception of their physical and mental conditions in relation to the act of teaching and learning [19,20]. As for the management of the socio-educational well-being of the university student and its relationship with the characteristics of the classroom, the educational institution, must reconcile the spatial binomial with the content of the subject [21]. For their part, the designer, the teacher and the students themselves will contribute through the appropriation and territoriality of the classroom to obtain positive results in terms of convenience, comfort and satisfaction [22,23].

The purpose of this study is to analyze the research trends on the impact of the university educational space on the well-being, motivation and social interaction of the student, considering the physical-environmental, socio-perceptual and motivational attributes.

In relation to the review of the literature carried out, and the work found that addresses this topic, the research problem concerns whether the design of the university educational learning and teaching space influences the socio-educational well-being of the student.

As regards the main limitation found in this research is to discern whether, among other variables, the number of publications relates to a certain regulatory regulation, is due to the requirements of interest groups, or, on the contrary, to the needs demanded by the education system itself.

Therefore, the principal objective of this research is to evaluate research trends on the impact of university classroom design on the socio-educational well-being of students globally during the period 2004–2018, considering the physical-environmental, socio-perceptual and motivational attributes.

To get responses to research issues, a sample of 1982 articles from a selection of scientific journals from the Scopus database has been evaluated. This review applies the bibliometric method to synthesize the knowledge base on the impact of the university classroom on managing the socio-educational well-being of the student. The results revealed contributions in this field of research, so that it has made it possible to identify the main drivers, their potential trends, and reveal gaps in critical knowledge. Thus, it can be assumed that, at present, university learning spaces require a transformation that positively links the socio-educational well-being of the university student with the classroom where the teaching act is practiced and learning.

Finally, it should be noted that among the lines of research currently being established in relation to the topic of the study, these relate, inter alia, to comparing the effect of the intrinsic characteristics of the university classroom on stress both in public and private universities, and in different countries; in addition to different studies that analyze the outcome in terms of inclusion and diversity.

## 2. Literature Review

The study of the impact of the university classroom on managing of the socio-educational well-being of the student is supported by the analysis of the main theory that together with the basic concepts define the framework of reference in this issue of research. In this way, explanatory theory defines how a set of phenomena behave, in order to generalize and perform a separate generalization of cases.

### 2.1. Framework

The literature review has allowed the detection and analysis of empirical, theoretical, critical, analytical or methodological scientific documents on the subject of research. The objective of this review was to obtain the research problem and the purpose of the study, in addition to generating a framework. Therefore, the literature analysis has offered publications that respond to the empirical study in the university classroom worldwide and has determined the impact factors of the classroom on the motivation and socio-educational well-being of the university student. The main publications in relation to the purpose of this study have made it possible to define in a concise and balanced way the definitions, concepts and theory on the impact of the university classroom in the management of the socio-educational well-being of the students.

Table 1 represents the leading results of the impact of the university classroom on the managing the socio-educational well-being of the student. For each contribution the title, the authors, the year of publication and the journal where it was published are indicated.

Otherwise, the revised literature provides definitions for the basic concepts of this research topic. Hence, it includes some reflections on the terms and concepts used in the context of this research.

The link of the university classroom with the management of the socio-educational well-being of the student must be established according to the axioms of the well-being theory or the PERMA model (Positive Emotion engagement, positive Relationships, Meaning, and Accomplishment/achievement) of Seligman, in 2011 [31]. It is an unforced choice theory, that is, it is a description of the free choice of the individual to increase the well-being. This state depends both on positive emotions and commitment, as well as on positive bonds and achievement. Thus, the individual should encourage the factors with which she/he identifies and feels comfort. Each element of the PERMA model must contribute to well-being and must be defined independently of the other variables in the model. Accordingly, all elements contribute allow to define well-being, such as the combination of feeling good and making sense in some activity, as well as maintaining good interpersonal relationships and having attractive goals so that they can become achievements [15,32].

The well-being theory, in line with positive psychology, relates to the state of flow, referring to an intense concentration, the flexibility to react to new problems, the maximum performance of the capacity of the individual, and the feeling of pleasure and happiness, derived from the activity performed [31,32].

In order to define the concept of socio-educational well-being in the context of research, the term of well-being, as an abstract concept, refers both to the psychological experience of pleasure and happiness, as well as to the state of satisfaction and tranquility that individual submits because of their good physical and mental conditions [33,34,35].

Likewise, well-being focuses on capacity development and personal growth, as indicators of positive functioning. In other words, well-being encompasses a series of sensations that allow an individual to judge her/his life globally [36,37]. Other studies also positively relate stress coping responses; while other studies highlight the proportional relationship between personality and well-being, or between life goals, self-perceived satisfaction and well-being [31,38]. Similarly, the strategies aimed at addressing and solving problems are related to high well-being, while an unproductive style, by contrast, is related to low well-being. In this sense, personality contributes to the self-perception of well-being in different vital areas, and, in a general way, well-being is related to variables such as age, sex, socio-economic status and ethnicity [36]. Regarding the factors that define well-being, self-acceptance, mastery of the environment, personal growth, self-efficacy, positive relationships with other individuals (implies the capacity for empathy), autonomy, and having a purpose that makes sense stand out to the life [36,38].

This concept transferred to the educational context has a special relevance. Thus, well-being in the environment is related to improving the teaching and learning process, increasing the capacity for attention and concentration, and promoting creative and holistic thinking [38,39]. In particular, well-being in the classroom refers to the development of assertiveness in relation to the increase of individual security when it comes to giving their opinion, to the ability to solve problems and conflicts, to decision making considering the advantages and disadvantages, the development of resilience in terms of the ability to establish strategies to recover well-being in adverse situations [37]. For these reasons, the classroom must allow the student to detect their personal strengths (honesty, perseverance, creativity, knowledge, loyalty or equanimity), and the achievement of achievement in relation to individual skills and the effort made in achieving a goal [31,37,40]. The integration of the student, as an absolute participant in the classroom environment, allows him to achieve well-being, in terms of connecting with space, and this has an impact on positive feelings, efficiency, interior order and external connection.

Notwithstanding the above, social welfare includes the factors that participate in the quality of life of the individual, so that they allow their tranquility and satisfaction. It is an unobservable condition directly, which is understood and can be compared between different spaces from reflections. Thereby, the term socio-educational adds the goal of education to the concept. In this way, the concept of socio-educational well-being of student is a key factor in achieving better levels of care, success and motivation. While, historically, academic classrooms have not considered the parameters of comfort and well-being, it is now an analyzed factor, which links the learning space with student behavior [39,41].

The concept of personal space, studied by Hall in 1966, refers to the interpersonal distance that helps and allows the individual to interact with peacefulness and this is influenced by culture [42]. In 1975, Altman integrated into the spatial conduct model concepts related to behavior and functions attributed to personal space, such as appropriation, privacy, territoriality and overcrowding. In this manner, individuals not only respond to environmental or physical conditions, but also take steps to influence, modify and restructure their environments [43,44]. From this perspective, the university classroom becomes a space for interaction, where students make sociocognitive exchanges with their peers, as well as strengthen the development of their personality traits [22,45,46]. Thereby, students and teachers create emotional links, positive or negative, with architectural buildings. The sense of belonging becomes relevant, because the institution of higher education becomes, together with your home and neighborhood, the next environment where your experiences will be given, and the identity of place arises [28,42,44].

Personal space is understood as a mechanism that regulates the boundaries between people and as a resource of alert to the invasion of space by another individual. Hence, it fulfills two fundamental roles, of self-protection and as a regulator of privacy [16,38,47].

Thereby, the concept of the university classroom refers to the educational space where the discents live, coexist, knowledge is transferred and, therefore, they are formed as citizens, with critical capacity, values and as protagonists of society. Nowadays, learning spaces are not understood as a simple volumetric container of activities, but their concept goes beyond a mere architectural object. This, being appropriated by students and teachers, manages to influence academic motivation, behavior and social relationships [2,38,43,48]. Thus, although so far, there is no single model for defining an optimal learning space [45,46], the physical environment of the classroom is considered as one of the most important indicators that determine benefits in student learning [36,40,43].

### 2.2. Impact Factors of the University Classroom on Socio-Educational Well-Being

**Figure 1:**
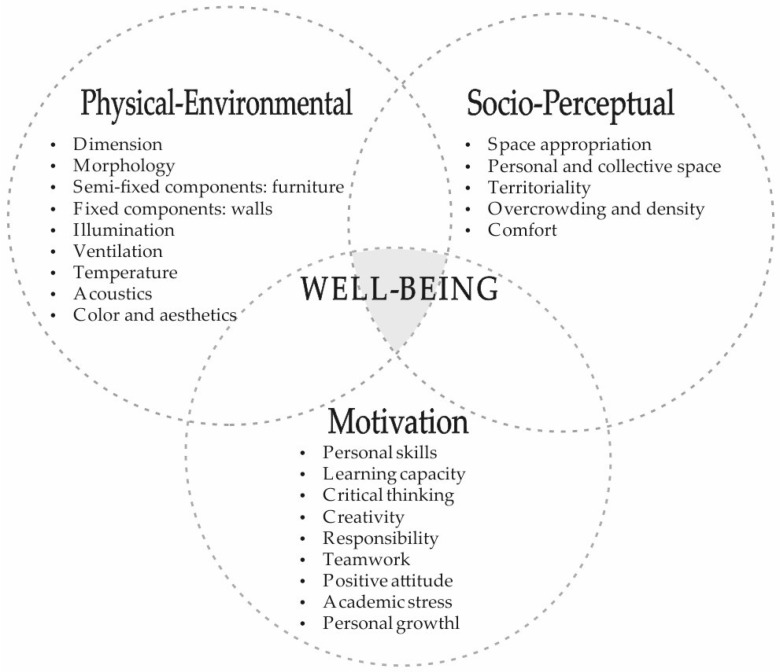
Conceptual structure and dimensions of the impact of the university classroom on managing of the socio-educational well-being of students.

The revised literature recognizes certain design factors involved in the classroom and their level of impact, both on the motivation [2,43,49,50] and on the social relations of the students [28,38,45]. The impact factors of the university classroom on managing of socio-educational well-being are grouped into three dimensions: physical-environmental, socio-perceptual and motivational. This classification attends previous studies [1,2,24,27,28,36,38,40], from which the concepts, theoretical reflections and practical studies are based, in order to translate them into quantifiable dimensions and indicators. Thus, the attributes of the learning space involved in the academic act are grouped into physical-environmental, socio-perceptual and motivational. Accordingly, Figure 1 shows the conceptual structure and dimensions of the university classroom’s impact on managing of the socio-educational well-being of students.

In 1995, Göttler expressed the influence of the physical and environmental characteristics of the educational environment on the social interactions and other psychosocial aspects of the student. Since then, numerous authors have continued with the approach to the issue from a psychological and physical approach [1,2,17,28,40,48,51].

In recent decades, this research topic has become particularly relevant with the publication of a large number of papers worldwide [1,17,24,27,28,36,40]. It should be noted that the changes experienced in the educational and social field have been reflected in the growing interest in knowing the variables involved in the academic act. These have addressed the relationship between the attributes of the physical space, the methodology and the influence on the teaching and learning process and students [26,43].

The design, quality and adequacy of learning spaces encourage students to maintain positive emotions, feel integrated and, thereby, to experience more favorable academic results [28,49,52,53]. Academic achievement is influenced by the articulation between the physical and architectural conditions of the building, and the social and perceptual environment that students appreciate from them, impacting their performance and motivation [50].

The literature shows evidence of the link between student satisfaction with the environment and the academic results obtained. Consequently, if the student experiences personal well-being and attachment to where she/he spends much of her/his daily life, this results in a positive impact on the attention, motivation, learning and sociability [40,54].

On the other hand, the impact of design on learning spaces, considering that the space intervenes in the social connection of students, thus encouraging collaboration, reflection, exchange and interaction. Conversely, if the design is insufficient, it can promote the development of childhood disorders, such as tacit muteness and lack of social interaction [40,50].

The influence of learning environments on children’s cognitive development and early literacy is undeniable. In such away, the primary cognitive development of human beings occurs through the relationships we maintain with our environment and the sociocultural stimuli perceived as external information. Hence, the study of the relationship between physical space and its impact on human behavioral processes is not novel [44]. Learning spaces are affected by variables of very different nature, in particular the physical, environmental, technological and social type. Therefore, determining its impact on those who inhabit it is complex.

Literature indicates that the physical attributes of the environment and the configuration of the learning space act on the perception of students. In this dialogue where the level of well-being and the functional possibilities offered by space are related, the impact on the learning process of those who inhabit it can be positive if the conditions are [30,38,40,55].

Learning is a multi-causal process that requires the integration of physical and environmental conditions to generate an enabling climate, allowing students’ behaviors to be more assertive and school environments to be healthy and rewarding [40,50]. The physical-environmental dimension establishes the relationship between the physical factors or conditions of the environment and its influence on the development of learning processes. From a holistic perspective, a series of variables are determined that favor student stimulation according to the configuration and design of the classroom, considering parameters of the environmental design [1,27,36].

Thus, there is a relationship between the environmental variables of the built space and its effect on teaching and learning processes, such as lighting, temperature, thermal comfort, color, materials, noise level, indoor air quality [36,38,52,53,56]. On the other hand, the spatial dimension addresses the impact of classroom environments on student behavior, attitudes, and achievements. Thereby, the physical distribution of the school environment, including all the components that configure it, as is the case of spatial proportionality and the physical arrangement of the classroom, are considered as external conditions of learning.

In this way, spatial variables defined by the physical characteristics of the classroom act as a scenario. It underpins the social and motivational actions that the student requires, maintaining a reciprocal and complementary relationship [38,45,57]. Dialogue between human being and environment involves and interlinks various factors linked to each other making it necessary to conceive of the idea of a complex and holistic environment. Moreover, in relation to the impact that educational buildings generate on the attitudes and behaviors of students, the fixed and permanent structural elements allow to define the territory, in addition to conditioning and delimiting movement and behavior in the inside [17,52].

In this sense, the morphology of the building, the size, the enclosures, the floors and ceilings and internal divisions of the classrooms must offer a visual continuity, supported by methodologies that favor participatory, active, collective and collaborative. Thus, students better perceive group cohesion, commitment to tasks and cooperation when the organization of seats is in small groups and allows the visibility of the rest [36,47]. This allows them to easily interact with peers, and group discussions and activities are favored. In this way, flexibility and functionality are qualities that design professionals seek, in order to generate open spaces that promote collective work and promote personal relationships [24,26]. These premises seek to prioritize the design, construction and use of university classrooms, which together with the harmonious performance of pedagogical discourse making, together with teachers, the environment become the third teacher [2,27,38].

The socio-perceptual dimension considers the subject as an active protagonist that inhabits the school space, and the impact on her/his behavior. The analysis focuses on human action and interaction in spaces, an aspect that addresses Environmental Psychology [24,38,45]. Accordingly, the perceptual variables of the classroom integrate the concepts related to the indicators of behavior and the concepts of personal space, privacy, territoriality and overcrowding of the subject.

In this sense, the location within the classroom is decisive in the perception of the student, while the preference of the company with friends generates positive differences in motivation and social relationships [42,58]. On the other hand, the views towards an attractive landscape act as inspiration, so that the interaction of the person with the environment favors the development of cognitive and emotional abilities [42]. There is a reciprocity in the transformative relationship between individual and environment. Subjects are influenced by their behaviors, emotions and experiences through the environment, but at the same time, individuals are protagonists in their modification.

In 1978, Canter, a precursor to the psychological perception of the classroom and the degree of satisfaction provided by a school space in the subject, noted that “by inhabiting the classroom, the individual is involved in the physical experimentation of his architectural space, all of this, taking into account the responses to certain variables” [30,56]. In this line, around the 1980s, a change of direction took place and new theoretical and methodological approaches emerged, giving way to perspectives focused on social and collective phenomena in relation to the physical socio-environment.

From this perspective, the classroom is not only a space where the students live, but it is also the place that represents the family, the activities, customs and culture to which the person belongs [42,44], and defines her/his identity. These aspects, together with the identity of the place, personal space, privacy, territoriality and overcrowding, intervene in the perception that people form of the learning space. Therefore, concepts arise such as place-identity [59], which refers to the place identity as a substructure of the identity of an individual’s being; and place-dependence [59], referring to the link between the person with a particular place. In these cases, subjective components that depend on two variables come into play: the quality of the place in question and the comparison of that quality with that of other places. All this transferred to the educational space, suppose a bond of social and affective contention between those who share it, and be considered as the identity basis of the group.

Finally, the motivational dimension emerges from other variables misused in research, such as academic performance. This dimension serves cognitive and attitudinal factors that influence student efficiency [28,37,55]. In this way, teaching methodologies for processing information, the responsibility of the student towards learning, and the presence of social networks as a transformative element of human behavior in relation to distraction are recognized, communication, emotions, autonomy and identity. Academic stress is also a factor of concern for assessment and failure [30,60].

Motivation influences learning, so the design of the university classroom must improve motivation in learning environments. For example, Keller’s ARCS model (an acronym for Attention, Relevance, Confidence and Satisfaction) explains its relationship to learning processes [61,62], in relation to motivation theory.

Digital technologies applied to training and education also enhance motivation in teaching and learning processes and offer new opportunities for learning [63]. The results indicate that teachers attribute high potential to these technologies to enrich collaborative work activities among students, as well as to achieve the development of cross-cutting skills. Social relationships are also favored, multiplying their effect when the student is willing to strive.

## 3. Methodology

In order to achieve the objective proposed in this study, bibliographic data has been analyzed using two methods. A systematic (qualitative) and a bibliomeric (quantitative) analysis of the data has been carried out.

### 3.1. Bibliometric Method

Scientometric is recognized as the scientific study of science and its results and is based primarily on the works of Solla Price and Garfield. In practice, there is a significant connection between scientometric and other scientific disciplines: bibliometric, information system, information science and scientific policy [64].

Likewise, bibliometric is a component of the scientometric that utilizes mathematical and statistical processes to the scientific production and the authors that generate it, with the purpose of researching scientific activity. The instruments applied to measure aspects of scientific activity are bibliometric indicators; these are measures that offer evidence on the results of scientific activity [65,66]. It was pioneered by Garfield in the mid-20th century and has since become prevalent in scientific research and has contributed to reviewing knowledge in several disciplines. Hence, scientometric together with bibliometric has evolved from reflection on scientific development and the availability of various databases for the researcher.

The purpose of this study is to recognize, organize and analyze research trends in the impact of the university classroom on managing of the socio-educational well-being of students, considering the physical-environmental, socio-perceptual and motivational attributes. To accomplish the proposed purpose, a quantitative analysis has been implemented, using bibliometric. In latest decades, it has provided to the review of scientific knowledge, and has been used productively in various scientific disciplines: health, engineering, economics, administration, education or ecology [67,68,69].

The methodology has been developed to analyze the scientific communities associated with this theme. The relationships between authors, institutions and countries, interpreted through the co-authorship of each document, have been analyzed, as well as analyzing the relationships between the keywords of all articles based on co-occurrence [70].

The co-citation analysis allows the observation of documents with citations and references cited, which can show the intellectual basis and trends in a particular field of research. Thus, the authors, institutions and countries are determined based on the co-citations of the rest, which represent relevance in this discipline, so that these generators of scientific production can be substitutes for the ideas they represent.

In this line, the co-occurrence analysis is used in order to provide a graphic visualization of the interconnection of the key terms within the documents analyzed. Generally, co-occurrence networks are used in order to facilitate a graphic visualization of potential relationships between authors, institutions, countries or terms in a text. Thus, the proximity relationship of two or more terms in a text unit can be observed, so that, if the terms co-occur in a sentence, there is a probability of their semantic relationship [71].

In short, the co-occurrence criteria allow revealing and grouping strongly related concepts within the set of documents or records. This procedure examines documents in order to look for two or more concepts that tend to be presented together.

The indicators of the collaboration structure, which measure the links between the authors, institutions and countries, have been analyzed through the processing tools and network maps due to their reliability and suitability in the bibliometric analysis [72].

### 3.2. Data Collection

Through this methodology, the interest in the subject matter of our study has evolved, by modifying the most relevant authors, countries, journals and keywords in recent years. Several databases of scientific papers related to the subject that have been studied have been consulted.

The two large scientific databases, Web of Science and Scopus, raise the main problem of the comparison and consistency of statistics derived from different data sources. It has been shown that Scopus has more indexed journals than Web of Science, in addition to minimizing the risk of losing documents during the search. Among its advantages, it stands out that it is easily accessible and offers some tools for viewing and analyzing data, as well as the option to download content in different formats, and that it provides a variety of data for each selected publication, its analysis and the comparison between them [73].

The Google Scholar database has not been taken into account, as it has some limitations. In this sense, it includes a greater amount of non-relevant variables, so that the cleaning of the data is more laborious, the processing and classification of the results require more effort, and includes a large number of articles not reviewed by peers, that is, it contemplates publications with a low quality level.

For these reasons, the information received from the Scopus database of Elsevier has been selected, as it is the largest repository of scientific articles and with a greater number of journals and authors, with peer review, compared to the rest of the databases [74]. In addition, this presentation in greater detail in the treatment of the information corresponding to each author, institution and country, of those consulted.

The method used was to carry out a complete search on the Scopus database, applying a search string, applying Boolean operators to the terms that combine this research: “higher education”, “university”, “classroom”, “well-being”, “integration”, “socioeducational” and “management”.

A descending search has been carried out in order to study the topic of research. With this type of search, first a sample of data from a broad general topic is selected and, sequentially, more restricted searches of the initial sample are performed, in order to define the data of a specific topic. Moreover, data abstraction consists in reducing a particular set of data to a simplified representation, that is, it refers to the process of removing characteristics from a research field to reduce it to a set of particular characteristics.

Accordingly, in a first search, the key concepts extracted from the review of the literature were included, including the entire time horizon, that is, from the publication of the first document on thematic study until the last full year, that is, from 1944 to 2018. In addition, all types of sources were included, according to the Scopus database (article, review, book, book chapter, conference paper, conference review, letter, editorial, note, short survey, business article or press, erratum and data paper). This search yielded a total of 4379 documents. Subsequently, the time horizon was limited to the last fifteen years (2004–2018), a period where the study presents relevance, including all types of sources. This second search yielded a total of 2389 documents. Finally, in the third search, only the articles were selected. The decision is based on the fact that the articles are the only documents submitted to a peer review process, which guarantees the scientific quality of the works. This last search yielded 1982 documents.

**Figure 2:**
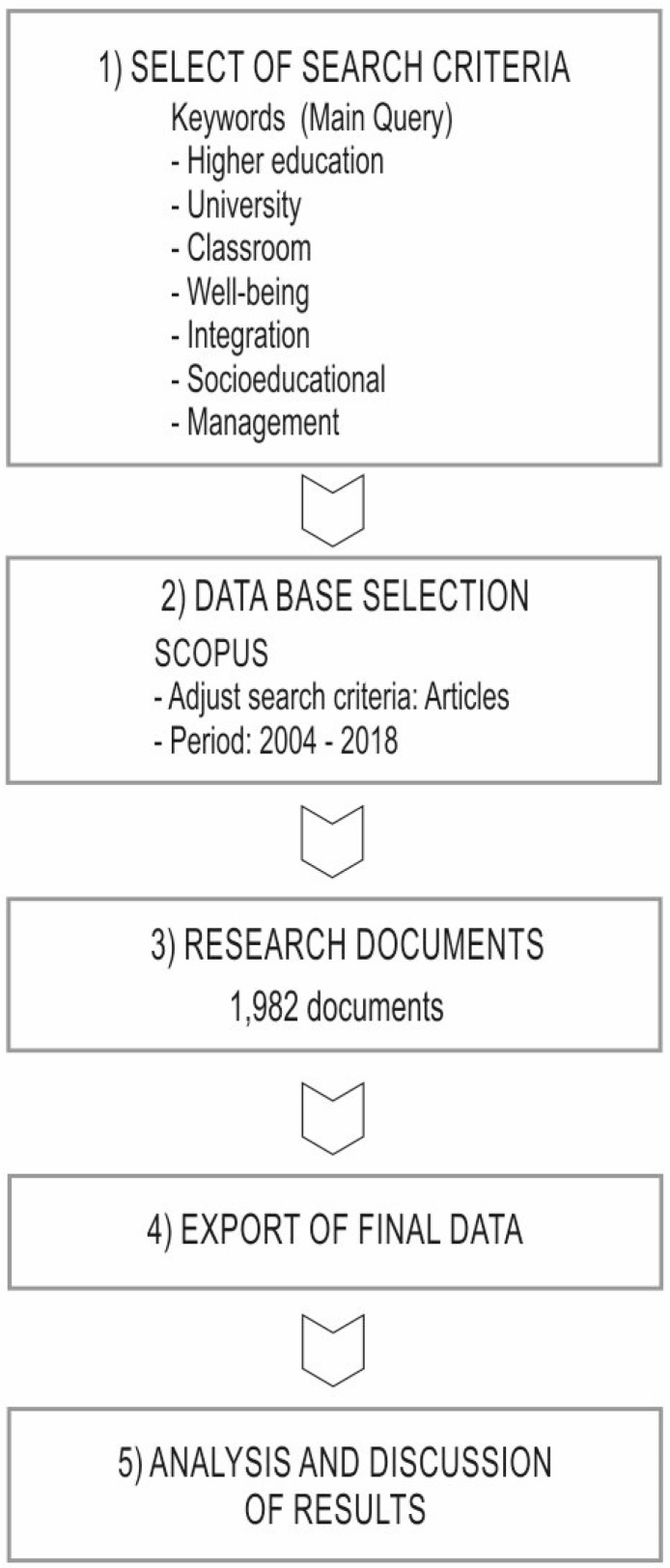
Flowchart of the protocol followed in the selection of documents.

In such manner, the purpose was to analyze the subfields of the title, abstract and keywords over a period of 15 years (2004 to 2018), as reflected in other bibliometric works [75,76]. The sample of articles examined was obtained during a search in November 2019, which included only scientific articles, in open and non-open access. Thus, the final sample included a total of 1982 documents. The variables analyzed were year of publication, journal, author, thematic area, country of affiliation of the author, institution where the author is associated, and keywords that describe the scientific publication. Figure 2 shows a scheme with the steps followed in the methodology applied in this study.

VOSviewer (version 1.6.10., Leiden University, Leiden, The Netherlands) is a software tool for keyword processing and grouping analysis used for map visualization, which allows grouping by co-authorship and co-occurrence. Additionally, using the VOSviewer tool, the collaborative structure indicators, which measure the links between authors and countries, have been studied through network mapping and processing instruments due to their reliability and suitability in the bibliometric analysis, as well as for the identification of research trends based on the use of keywords [77,78,79]. The results obtained from the evaluation of scientific production in this research topic are valuable for academics, researchers in the area of health and other managers of HEIs.

## 4. Results and Discussion

### 4.1. Scientific Production

Table 2 indicates the evolution of the most important characteristics of published articles on the impact of the university classroom on managing of the socio-educational well-being from 2004 to 2018. In this period, interest in the subject of research has increased, particularly in the last 6 years, as seen in the variables evaluated. Thus, if in the period 2001–2003 135 articles on this issue were published, in the 2016–2018 period the total amounted to 698, that is, 5.17 times more. The growth is particularly accentuated in the latter three years, where 35.20% of the total articles published in the period analyzed have been published, and 37.30% of the authors contributed. Thereby, 2018 is the year which more publications generated, with 301 articles.

**Figure 3:**
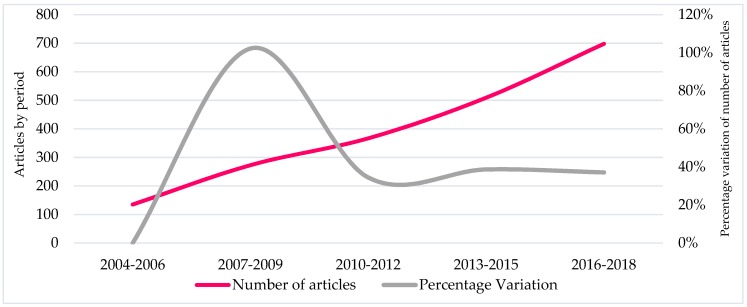
Progression of the number of articles and percentages of variation between triennials.

Figure 3 shows the evolution in the number of articles and their percentage of variation between each triennium analyzed. Additionally, to the substantial increase in the number of articles published in the last 6 years, the percentage growth produced in the second period studied (2007–2009) with 102.20% stands out. This percentage growth in the number of publications is because it is the first three-year period in which 200 articles are exceeded (273), and includes the first year, 2009, with more than 100 articles published (106).

In the same way as with the articles, the total number of authors has also increased for the period analyzed. In the last triennium, 2016–2018, 37.30% of the total authors of the 15-year period are concentrated. It is noted that the number of authors who published in this topic of research between 2004 and 2006 was 289, amounting to 1986 authors in the triennium 2016–2018. This is a larger increase than experienced in the number of articles published, because the average number of authors by article has also increased. Thus, in 2004–2008 the average number of authors by article stood at 2.1 authors by article, while in the last period (2016–2018) it increased to 2.8 authors by article, with a maximum of 3.1 in the fourth period (2013–2015).

On the other hand, the number of countries involved in the publication of articles on this topic of research has increased from 43 in the 2001–2003 triennium to 179 of the last period analyzed. Throughout the period analyzed, the total number of countries that have contributed to the publication of articles on the impact of the university classroom on managing of the socio-educational well-being of students amounts to 134.

In addition, the number of citations increased from the first period (2004–2006) with 3,011 to the third triennium analyzed (2010–2012) with 4972. Since this trienium, the total number of citations of the total articles has been decreasing, with 3705 in the following period (2013–2015), and with 1257 in the last period (2016–2018). This circumstance is due to the fact that the published articles, that is, those corresponding to the last 6 years, will receive a greater number of citations in the coming years, for their recent publication and impact, in addition to their distribution in open-access [80], and this situation is related to the average annual number of citations by article. Thus, this average has been decreasing from 22.30 in the first triennium (2004–2006) to 1.80 in the last period (2016–2018).

The number of journals that published articles on the subject of study increased from 127 in the first period, 2004–2006, to 535, in the last triennium analyzed, 2016−2018. Likewise, the number of institutions increased from 215 in the 2004–2006 period, to 1296 in 2016–2018.

Finally, the number of references increased from the first period (2004–2006) with 3547 to the last three-year period analyzed with 24,899. This assumes that the average has been increasing from 25.61 in the first triennium (2004–2006) to 35.67 in the last period (2016–2018).

In this regard, Table 3 shows the 20 most cited articles in this field of research during the period 2004–2018. These findings are in line with the interest of research, in recent decades, to study, as main issues, the problem of educational failure, attitudes of rejection of traditional learning, and, consequently, with the demotivation of the student, marked by geographic, ethnic or gender variables [43,81,82]. On this side, scientific production is a participant in the search for solutions and in providing management elements to the educational environment to link achievement and well-being with the learning space [36,46].

### 4.2. Publications by Subject Area and Journal

During the time horizon analyzed, 2004−2018, there are several categories where work related to the impact of the university classroom on managing of the socio-educational well-being of students has been found. Thus, according to the Scopus classification, there are a total of 27 thematic areas in which the 1982 articles analyzed are classified. It is necessary to clarify that the same article can be classified in more than one category, depending on the interest of the author and the publisher.

**Figure 4:**
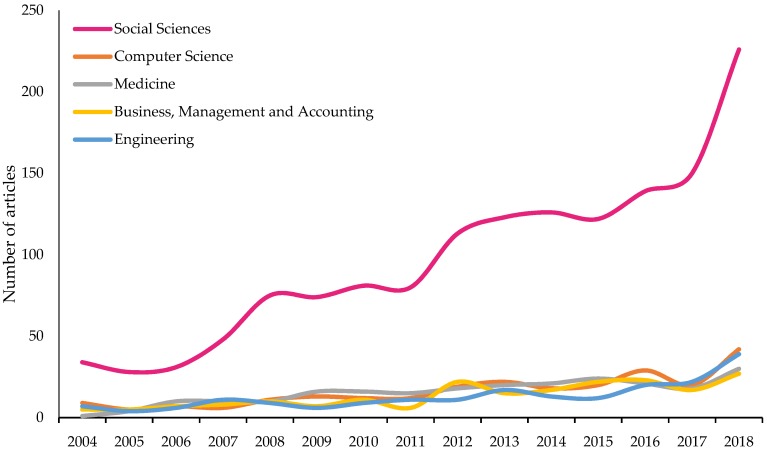
Growth trends of the main subject areas on the impact of the university classroom on managing the socio-educational well-being (2004–2018).

Figure 4 shows how the thematic classification of articles on the subject of research has evolved in the period 2004 to 2018. The Social Sciences category is the outstanding throughout the period studied, with 45% (1450) of the published articles on the topic of study. It is followed by the Computer Science category, with 8%. Medicine (7%), Business, Management and Accounting (6%) engineering (7%) are the following categories in order of importance. Therefore, the 5 most important categories represented in Figure 3, represent 73% of the documents published in this field of research from 2004 to 2018. Except for the Arts and Humanities (6%), Psychology (5%), and Nursing (4%) categories, the rest does not reach 2% of published works.

The association of publications in this field of study, mainly, to the Social Sciences category makes sense, since the sustainability factors of higher education relate to learning opportunities in the classroom university [83], or the emotional regulation of teachers and classroom management [84].

Table 4 displays the characteristics of the articles of the main journals in the publication on the topic of research. In the selection of the 20 journals with the highest number of articles published about research, the high percentage (40%) journals belonging to the first quartile of the SJR index, SCImago Journal Rank 2018. Furthermore, over the years, the topic of the link between the impact of the university classroom on managing of the socio-educational well-being has been interested in more journals and more authors, as evidenced by the growth in the number of articles and the variety of journals concerned.

By country, among the 20 most important journals are those of European origin: United Kingdom (7), Germany (1) and Ireland (1), which are also the journals that have a better position in the SJR 2018 ranking, and those of American origin, United States (6) and Canada (1).

The journals that have published most articles on this field of research have been Computers and Education (31), Teachers College Record (27) and Asia Pacific Education Researcher (22), so these journals represent 4.04% of the total articles published since 2004–2018. Computers and Education stands out because it concentrates a great interest in the scientific community, as evidenced by the high number of citations that concentrate its work, 976, and for the average number of citations by published articles, with 31.48 citations by article. Additionally, it is also the journal that presents the largest H index for published articles on this topic of research, 19, although it is quite far from the overall H index of the journal, for all subjects, which stands at 149. It is also the journal with the highest SJR impact factor: 2.323 (Q1), followed by Journal of School Psychology, with 1.751 (Q1), and Teaching and Teacher Education, with 1.512 (Q1).

The importance attached to the relationship of the university classroom with socio-educational well-being for the most productive thematic areas. Thus, Social Sciences includes journals such as Teachers College Record, Asia Pacific Education Researcher, Theory into Practice or Australian Journal of Teacher Education; while the area of Computer Science, contains Computers and Education, International Journal of Emerging Technologies in Learning, Australasian Journal of Educational Technology or International Journal of Engineering Education. These findings are linked to the factors or variables described in the literature review. The journals contained in the thematic area of Computer Science assess the physical variables of the classroom [56,60]; while the journals that publish thematic articles contained in the thematic area of the Social Sciences, consider mostly the socio-perceptual or motivational variables in their work [50,57].

### 4.3. Productivity of Authors, Institutions and Countries

Table 5 presents the main variables of the articles of the 10 most prolific authors in the publication on the impact of the university classroom on managing of the socio-educational well-being of students during the period 2004–2018.

The author who has published the most articles on the subject of research is the American, Reddy, from Rutgers University, with five documents, followed by a group of authors with four documents each: the Americans Bradshaw, Dudek, Vazou and Webster, Chai (Hong Kong), and Hudson (Australia). However, the author with the highest number of citations on the research study is the American Bradshaw, with a total of 125. Furthermore, he is the author with the highest average number of citations by article, with 31.25, followed by Chai (Hong Kong), with 76 citations and an average of 19 citations by article. Of the 10 most prolific authors in this field of research, six—Reddy, Bradshaw, Chai, Dudek, Vazou and Webster—have the highest H index, with 4.

It is noteworthy that the 10 most prolific authors in the publication of articles on this subject of research have American origin (6), followed by Hong Kong, Australia, Spain and Greece, with one each one. In addition, four authors published a final paper in 2018, the last year analyzed in this study, and three authors in 2017, indicating the importance and interest of the research topic.

**Figure 5:**
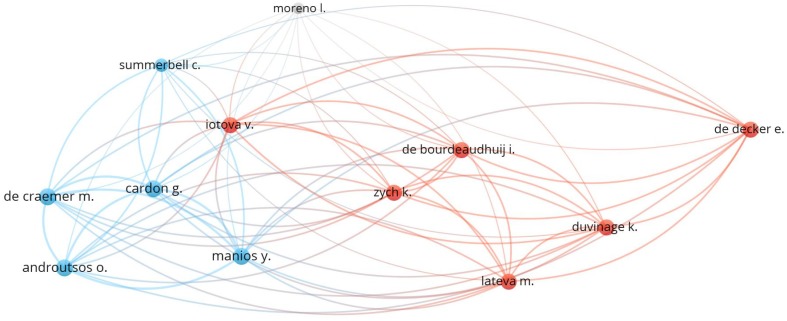
Network of cooperation based on co-authorship between authors (2004–2018).

Figure 5 shows the map of collaboration among the main authors who have published on the research study, based on co-authorship. Different colors represent the different clusters formed by workgroups in articles production, and the size of the circle varies depending on the number of articles by each author. The main authors are grouped into two clusters. Cluster 1 (red color) presents the collaboration between Zych, Lateva, De Bourdeaudhuij, De Decker, Iotova and Duvinage. While cluster 2 (blue color) consists of Androutsos, Cardon, De Craemer, Manios and Summerbell.

These results are in line with cooperative learning in university classes, as well as communication of teaching experiences and expectations in educational policies and classroom practices [89,98].

Table 6 presents the 10 most productive institutions in the publication of articles related to the research topic. The United States, with seven institutions, is the country with the largest presence in this ranking. Among them, Iowa State University ranks first, with 12 articles. The University of Virginia is the institution with the most citations in the articles in this research topic, with 466, and an average of 38.33 citations by article during the period 2004–2018. The University of Texas at Austin is the institution with the highest H index, with 8. It is noteworthy that, of these 10 institutions, six have published on this subject in 2018.

Table 7 lists the main variables of the countries with the highest scientific output on the field of research during the period 2004–2018. First, there is the United States, with a total of 787 articles and with the highest total number of citations, with 9496, that is, an average of 12.07 citations for each article on the subject of research, which represents the second highest average of citations by article, after the United Kingdom (12.81). The United States also has the largest H index, with 52. The second country with the highest number of articles is the United Kingdom, with a total of 154, and presents the second total number of citations, with 1973, and the H index, with 27. This peculiarity indicates the interest of American and English publications on the impact of the university classroom on managing of the socio-educational well-being [99,100,101,102]. The United States has been at the forefront of the ranking of the most prolific countries in the production of articles about research throughout the period analyzed, thus highlighting its research power. Hence, the United States, the United Kingdom, Australia, Spain, Australia and Turkey are the main drivers of research on the subject, having published 1263 articles, and representing 64% of the world’s total articles.

Table 8 shows the international collaboration of the countries with the highest number of works done. The United States is the country with the most articles published through international collaboration, with 46, with Canada, Australia, China, Taiwan, New Zealand, among its main contributors. It is followed by the United Kingdom and Australia, with 32 publications each one, Spain, with 28, and Turkey, with 26. The rest of the countries does not exceed ten articles with international collaboration.

**Figure 6:**
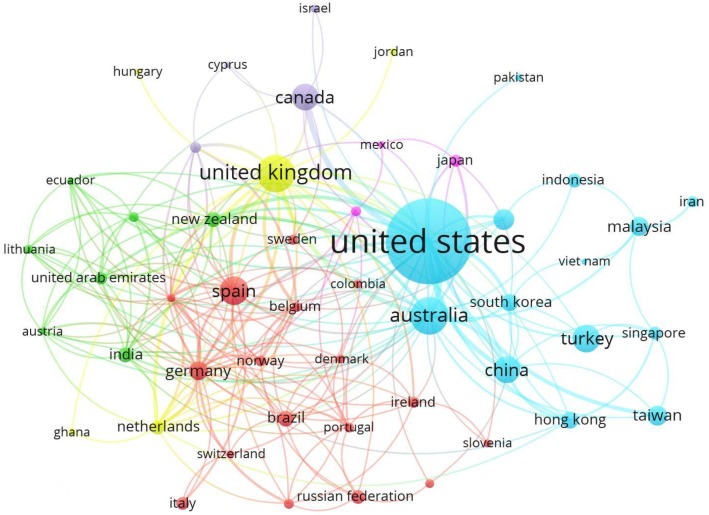
Network of cooperation based on co-authorship between countries (2004–2018).

Figure 6 shows a collaboration map between major countries based on the co-authorship of their authors. The different colors represent the different clusters formed by the groups of countries, and the size of the circle varies depending on the number of articles in each country. Thus, the greater the circle of each country, the greater the number of articles whose authorship it represents. Countries have been grouped into 6 clusters. Cluster 1 (red), the largest, includes 17 countries: Belgium, Brazil, Colombia, Denmark, Finland, France, Italy, Ireland, Norway, Germany, Poland, Portugal, Russian Federation, Slovenia, Spain, Sweden and Switzerland. Group 2 (blue), the largest along with cluster 1, is led by the United States, which shares works with Australia, China, Hong Kong, Indonesia, Iran, Malaysia, Singapore, South Korea, Taiwan, Turkey and Vietnam. Cluster 3 (green) is led by New Zealand, and includes Austria, Ecuador, India, Lithuania, Saudi Arabia and the United Arab Emirates. Cluster 4 (yellow) is headed by United Kingdom and includes Ghana, Hungary, Jordan and Netherlands. Cluster 5 (violet) includes Canada, Cyprus, Greece and Israel. Finally, cluster 6 (pink) consists of Chile, Japan and Mexico.

The impact and influence of the United States on the subject of study is well defined by both its scientific production and the cooperation in its publications. The United States, despite having the most powerful and influential HEIs at the international level, is a country that records a very high gap between the results obtained by those who did not finish secondary school and who have at least two years of university courses [97]. In addition, in the numerical skills assessment, American students were consistently below the OECD average. These results derive in the high interest in publishing articles to find the causes and solutions [49,99]. In this context, collaboration between countries in the publication of articles on the link between the university classroom and socio-educational well-being, mainly, is developed, among others, by scientific projects between different universities, conferences, symposia or scientific meetings [83,95,101].

### 4.4. Keyword Assessment

Table 9 lists the 20 most repeatedly used keywords in the 1982 articles on the impact of the university classroom on managing of the socio-educational well-being during the period 2004–2018. The relationship for the entire period is shown, as well as for the various three-year subperiods in which the considered time horizon of 15 years can be divided.

The terms “Human”, in 437 documents, and “Teaching”, in 346, occupy the first two positions throughout the period analyzed. They are followed by the term “Education”, in 332 publications, and “Students”, in 2031. On the other hand, the composition “Organization and Management”, is in fifth position, with 213 articles. For its part, the terms “University”, with 211 articles, and “Higher Education”, with 154, occupy the sixth and eighth positions, respectively. Also noteworthy are the terms “Well-being”, “Classroom” and “Integration”, among the 20 keywords in the period analyzed (2016–2018).

**Figure 7:**
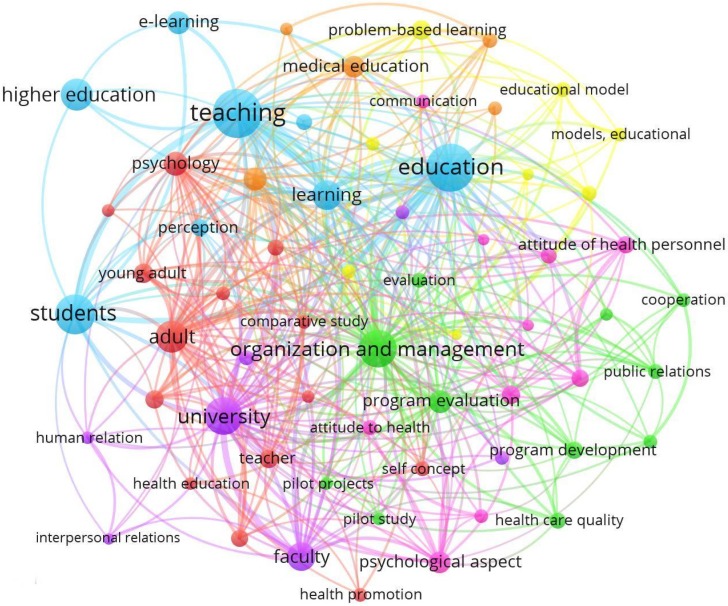
Keywords network based on co-ocurrence (2004–2018).

Figure 7 represents the network map for the keywords in the research articles on the research field for the period 2004–2018. The color of the nodes is used to differentiate the different groups or clusters according to the number of co-occurrences, while their size varies depending on the number of repetitions. Therefore, some lines of research developed by the different communities or groupings have been detected. Seven main lines of research are distinguished, which are grouped under the terms “Health Education”, “Program evaluation”, “Organization”, “Educational model”, “Motivation”, “Human relation” and “Procedures”.

These lines of research bring together all the concepts related to the topic of research, since it includes aspects related to the sustainability of institutions education in a globalized and connected world [103], and with the management that involves the active role of the institution [104].

As an additional advantage, it is noted that research on this topic continues to advance at the global level, with other concepts and strategies, such as “Knowledge Management”, “Education for Sustainability” or “Managing Behaviour” [105,106,107].

Organizations are also making an effort in line with education for sustainable development, since it is understood that, as UNESCO points out, education, in all its forms and all its levels, is one of the most effective tools for inducing necessary changes in order to achieve sustainable development and optimal management of socio-educational well-being [108,109,110,111].

The evolution of the keywords and their association in clusters is related to the dimensions or factors of the study theme indicated in Figure 1. Thus, there is a line of research that associates and links the terms related to the physical-environmental dimension (“integration”, “school buildings”, “innovation”, “technology integration”, “appointment”, “space”, “learning environment”, “accessibility”, “learning environment”, “large classroom”) [30,51], others with the socio-perceptual dimension (“perception”, “self-concept”, “human relation”, “inter-personal communication”, “interpersonal relation”, “self-efficacy”, “social behavior”, “collaboration”) [57,60], and finally, other keywords with the motivational dimension (“motivation”, “communication”, “decision making”, “leadership”, “satisfaction”, “attitude”, “collaborative learning”, “skill”) [39,56].

Other relevant terms have also been grouped around the line of educational management (“classroom management”, “educational model”, “personnel management”, “project management”, “learning management system”, “knowledge management”, “problem solving”, “problem based learning”, “marketing management”, “management in education”, “management information systems (MIS) students”, “management of innovation and change”, “management development”, “management and regulation of education”). On the other hand, the research is linked to the different thematic areas of Scopus, generating a map highlighting the terms “Education”, “University”, “Students”, “Higher Education” and “Organization and Management”.

## 5. Conclusions

The aim of this study was to study research trends on the impact of the university classroom on managing the socio-educational well-being worldwide over the previous 15 years, considering the physical-environmental, socio-perceptual and motivational attributes.

A bibliometric study of 1982 articles achieved from the Scopus database has been developed. Thematic areas, journals, authors, institutions and most productive countries have been recognized in publications on this field of research.

The number of scientific papers by year during the period 2004–2018 has increased, mainly in the last six years, where 1207 articles have been published, representing 60.90% of contributions on this research topic.

The Social Sciences thematic area is the most relevant, grouping 45% of the articles, and it is followed by Computer Science and Medicine, with 8% and 7%, respectively.

The most prolific journals have been Computers and Education, American Journal of Pharmaceutical Education, and Theory into Practice, with 4.04% of all articles published each one (34) in the analysis period. It should be noted that 40% of the journals that contribute to this topic are positioned in Scopus’ first quartile. Journal of School Psychology is the journal with the highest number of citations (420), which presents the highest H index for published articles on this subject area (8) and presents the best average number of citations by article, with 46.67.

The authors who have published the most about the field of research are the American Reddy from Rutgers University, with five articles, followed by a group of American authors (Bradshaw, Dudek, Vazou and Webster), Chai (Hong Kong), and Hudson (Australia), with four publications each one. Bradshaw, from the University of Virginia, is the author with the highest number of citations (125) and the highest average of citation by article (31.25).

The most prolific institutions in this area of research are the American ones Iowa State University, University of Texas at Austin and University of Virginia, and The University of Hong Kong (Hong Kong), and the University of Queensland (Australia), with 12 publications each one. The University of Virginia (United States) has the highest number of citations (466) and the highest average citations by article (38.83). In the group of the top 10 institutions with the most contributions to the subject of study, by country, the United States stands out with 7.

The main countries that have made an effort is this field of research, in order of importance are the United States, the United Kingdom, Australia, Spain and Turkey. Thus, the United States presents the largest number of published articles (787) and citations (9496). In relation to the countries that have made the greatest international collaboration in their articles, the United States, the United Kingdom and Australia stand out.

Nevertheless, this research has some limitations, which could be the basis for future research. Mainly, these come from the intrinsic characteristics of the quantitative analysis of the bibliometric method. One of these restrictions is that some authors publish few articles with high influence in a certain field of research. Furthermore, this method could be extended with other databases or quantitative or qualitative instruments, which would simplify a distinct perspective of the research. Alternatively, other types of documents, additionally to scientific articles, could also be incorporated in the study.

In the context of the limitations of this research, these refer to the breadth of the focus of the purpose of the study for university students, since their location, grade, course, gender, race or other defining characteristics have not been specified. In addition, another limitation refers to the use of a sample with quantitative data and not having considered the advantages of qualitative data. It is also noteworthy that the study addresses the possible metabias, particularly as parameters for study samples demographics were not delineated.

Future lines of research on this topic will focus on how neuroarchitecture analyses the impact that educational space has on learning activities, through holistic measurements and interpretations. Alternatively, other contributions should also study the impact of the interrelationship of the different natures of which the learning space is composed (physical, cultural, social, psychological, pedagogical, historical and human), in order to observe how their level of relationship may benefit or impede the notion of identity and the sense of belonging of the students. In addition, it should be explored how the transition between the intermediate level and higher education requires students to adapt to different processes and situations that require the implementation of various personal resources and assume participation in the new roles. This involves describing academic stress from the cognitive and affective processes that the student perceives of the impact of academic stressors.

In this regard, it is necessary that future research be directed, not so much to the description of the factors that impact the well-being of the student, since they are very developed, and to investigate in a practical way how specific modifications, for example physical space, have impacts on the motivation and well-being of university students. In line with the findings of this study, they can be referred to practical applications, such as visual thinking, flipped classroom and active cooperative learning methodologies that influence students’ communication and interpersonal relationships.

Finally, it should be noted that it has been noted that trends in research on the impact of the university classroom on managing of the socio-educational well-being of students worldwide have followed an upward trend and stabilized in optimal publication rates in recent years.

## Figures and Tables

**Table 1:** Main publications reviewed of the object of research.

Title [Reference]	Author(s)	Year *	Journal
Learning in and for multi-agency working [24]	Daniels, H.; Leadbetter, J.; Warmington, P.; Edwards, A.; Martin, D.; Popova, A.; Brown, S.	2007	Oxford Review of Education
Effects of school design on student outcomes [17]	Tanner, C.K.	2009	Journal of Educational Administration
Employee wellbeing in the higher education workplace: a role for emotion scholarship [25]	Woods, C.	2009	Higher Education
A collaborative approach to college and university student health and wellness [7]	Fullerton, D. S.	2011	New Directions for Higher Education
A study on student perceptions of higher education classrooms: Impact of classroom attributes on student satisfaction and performance [26]	Yang, Z.; Becerik-Gerber, B.; Mino, L.	2013	Building and Environment
Continuity and conflict in school design: a case study from Building Schools for the Future [27]	Tse, H.M.; Learoyd-Smith, S.; Stables, A.; Daniels, H.	2015	Intelligent Buildings International
School building condition, social climate, student attendance and academic achievement: A mediation model [28]	Maxwell, L.	2016	Journal of Environmental Psychology
The holistic impact of classroom spaces on learning in specific subjects [1]	Barret, P.; Davies, F.: Zhang, Y.; Barrett, L.	2017	Environment and Behavior
Happiness in Higher Education [29]	Elwick, A.; Cannizzaro, S.	2017	Higher Education Quarterly
Learning Space Design and Classroom Behavior [2]	Baum, E. J.	2018	International Journal of Learning, Teaching and Educational Research
Environmental factors affecting students’ stress in the educational environment: A case study of Shiraz schools [30]	Najafi, N.; Movahed, K.; Barzegar, Z.; Samani, S.	2018	International Journal of School Health

* Year: Year of publication of the article.

**Table 2:** Major characteristics of the articles of the impact of the university classroom on managing the socio-educational well-being (2004–2018).

Period	A	AU	C	TC	J	R	I	TC/A	AU/A	R/A
2004–2006	135	289	43	3011	127	3457	215	22.30	2.14	25.61
2007–2009	273	720	91	4801	224	7792	443	17.59	2.64	28.54
2010–2012	367	958	103	4972	312	11,878	658	13.55	2.61	32.37
2013–2015	509	1574	141	3705	430	17,867	1003	7.28	3.09	35.10
2016–2018	698	1986	179	1257	535	24,899	1296	1.80	2.85	35.67

A: total number of articles; AU: total number of authors; C: total number of countries; TC: total number of citations; J: total number of journals; R: total number of references; I: total number of institutions; TC/A: total number of citations by year; AU/A: total number of authors by year; R/A: total number of references by year.

**Table 3:** Most cited articles on the impact of the university classroom on managing of the socio-educational well-being (2004–2018).

Year	Title [Reference]	Author (s)	Journal	TC
2004	Treating Children with Early-Onset Conduct Problems: Intervention Outcomes for Parent, Child, and Teacher Training [81]	Webster-Stratton, C., Reid, M.J., Hammond, M.	Journal of Clinical Child and Adolescent Psychology	497
2010	Reducing the gender achievement gap in college science: A classroom study of values affirmation [82]	Miyake, A., Kost-Smith, L.E., Finkelstein, N.D., (...), Cohen, G.L., Ito, T.A.	Science	288
2012	Recent trends in research on teacher-child relationships [85]	Sabol, T.J., Pianta, R.C.	Attachment and Human Development	218
2015	Improvements from a flipped classroom may simply be the fruits of active learning [61]	Jensen, J.L., Kummer, T.A., Godoy, P.D.D.M.	CBE Life Sciences Education	215
2010	Real-world learning opportunities in sustainability: from classroom into the real world [8]	Brundiers, K., Wiek, A., Redman, C.L.	Int. J. Sustain. High. Educ.	205
2009	Saving time or innovating practice: Investigating perceptions and uses of Learning Management Systems [86]	Lonn, S., Teasley, S.D.	Computers and Education	190
2013	Looking at the Impact of the Flipped Classroom Model of Instruction on Undergraduate Multimedia Students at CSUN [62]	Enfield, J.	TechTrends	176
2014	Evidence for General and Domain-Specific Elements of Teacher-Child Interactions: Associations with Preschool Children’s Development [87]	Hamre, B., Hatfield, B., Pianta, R., Jamil, F.	Child Development	167
2007	Lexical bundles in university spoken and written registers [88]	Biber, D., Barbieri, F.	English for Specific Purposes	161
2008	Teachers’ views and beliefs about bullying: Influences on classroom management strategies and students’ coping with peer victimization [89]	Kochenderfer-Ladd, B., Pelletier, M.E.	Journal of School Psychology	158
2004	Constraints experienced by beginning secondary science teachers in implementing scientific inquiry lessons [90]	Roehrig, G.H., Luft, J.A.	International Journal of Science Education	154
2012	Higher education scholars’ participation and practices on Twitter [91]	Veletsianos, G.	Journal of Computer Assisted Learning	145
2009	Learning to BREATHE: A pilot trial of a mindfulness curriculum for adolescents [92]	Broderick, P.C., Metz, S.	Advances in School Mental Health Promotion	144
2005	The contribution of classroom setting and quality of instruction to children’s behavior in kindergarten classrooms [93]	Rimm-Kaufman, S.E., La Paro, K.M., Downer, J.T., Pianta, R.C.	Elementary School Journal	143
2013	Improving classroom learning environments by cultivating awareness and resilience in education (CARE): Results of a randomized controlled trial [16]	Jennings, P.A., Frank, J.L., Snowberg, K.E., Coccia, M.A., Greenberg, M.T.	School Psychology Quarterly	136
2012	The Use and Abuse of Cell Phones and Text Messaging in the Classroom: A Survey of College Students [94]	Tindell, D.R., Bohlander, R.W.	College Teaching	122
2006	Stance in spoken and written university registers [95]	Biber, D.	Journal of English for Academic Purposes	122
2014	A meta-analysis of blended learning and technology use in higher education: From the general to the applied [96]	Bernard, R.M., Borokhovski, E., Schmid, R.F., Tamim, R.M., Abrami, P.C.	Journal of Computing in Higher Education	119
2005	‘We do not seem to have a theory … The theory I present here attempts to fill this gap’: Inclusive and exclusive pronouns in academic writing [97]	Harwood, N.	Applied Linguistics	117
2004	Increasing preservice teachers’ self-efficacy beliefs for technology integration [63]	Wang, L., Ertmer, P.A., Newby, T.J.	Journal of Research on Technology in Education	117

Y: year of publication of the article; TC: total number of citations of the article.

**Table 4:** Most productive journals in number of articles on the impact of the university classroom on managing the socio-educational well-being (2004–2018).

Journal	A	TC	TC/A	H (A)	H (J)	SJR (Q)	C	A				
								2004–2006	2007–2009	2010–2012	2013–2015	2016–2018
Computers and Education	31	976	31.48	19	149	2.323 (Q1)	United Kingdom	1	13	7	5	5
Teachers College Record	27	274	10.15	8	78	0.995 (Q1)	United Kingdom	0	5	7	3	12
Asia Pacific Education Researcher	22	134	6.09	7	20	0.424 (Q2)	Philippines	0	2	7	8	5
International Journal of Emerging Technologies in Learning	19	29	1.53	4	15	0.219 (Q3)	Germany	0	0	2	4	13
Nurse Education Today	17	251	14.76	8	65	1.041 (Q1)	United Kingdom	0	7	2	4	4
American Journal of Pharmaceutical Education	16	192	12.00	8	52	0.630 (Q2)	United States	1	2	6	5	2
Theory into Practice	12	179	14.92	7	50	0.522 (Q2)	United States	0	6	0	2	4
Australasian Journal of Educational Technology	11	96	8.73	7	40	0.721 (Q1)	Australia	0	0	7	1	3
International Journal of Engineering Education	11	63	5.73	5	44	0.425 (Q2)	Ireland	4	3	2	0	2
Qualitative Report	11	12	1.09	3	24	0.410 (Q3)	United States	4	3	2	0	2
Australian Journal of Teacher Education	10	96	9.60	6	24	0.370 (Q2)	Australia	0	1	3	5	1
Educational Technology and Society	10	118	11.80	6	73	1.085 (Q1)	Taiwan	1	1	2	2	4
Education and Treatment of Children	9	38	4.22	4	36	0.550 (Q3)	United States	0	0	0	4	5
ELT Journal	9	144	16.00	5	47	1.020 (Q2)	United Kingdom	1	1	2	0	5
Higher Education Research and Development	9	265	29.44	8	37	1.294 (Q1)	United Kingdom	1	3	2	3	0
International Journal of Learning	9	11	1.22	3	10	0.130 (Q4)	United States	0	3	6	0	0
Journal of Advanced Oxidation Technologies	9	0	0.00	0	21	0.274 (Q4)	Canada	0	0	0	0	9
Journal of Nursing Education	9	10	1.11	2	57	0.585 (Q2)	United States	1	3	2	3	0
Journal of School Psychology	9	420	46.67	8	83	1.751 (Q1)	United Kingdom	0	2	2	5	0
Teaching and Teacher Education	9	247	27.44	7	104	1.512 (Q1)	United Kingdom	0	4	2	0	3

A: total number of articles; TC: number of citations for all articles; TC/A: number of citations by article; H(A): Hirsch index in articles of this research topic; H(J): Hirsch index in journal; SJR: Scimago Journal Rank (SJR indicator); Q: Quartile (quartiles, Q1 to Q4, refer to the classification of the journal within a subdiscipline using the SJR index); C: country.

**Table 5:** Most productive authors in number of articles on the impact of the university classroom on managing the socio-educational well-being (2004–2018).

AU	A	TC	TC/A	Institution	C	1st A *	Last A *	H Index *
Reddy, L.A.	5	57	11.40	Rutgers University	United States	2013	2017	4
Bradshaw, C.P.	4	125	31.25	University of Virginia	United States	2010	2016	4
Chai, C.S.	4	76	19.00	Chinese University of Hong Kong	Hong Kong	2009	2013	4
Dudek, C.M.	4	36	9.00	Rutgers University	United States	2013	2017	4
Hudson, P.	4	22	5.50	Southern Cross University	Australia	2009	2011	3
Vazou, S.	4	6	1.50	Iowa State University	United States	2018	2018	4
Webster, C.A.	4	14	3.50	University of South Carolina	United States	2010	2018	4
Androutsos, O.	3	24	8.00	Harokopio University	Greece	2013	2018	2
Asensio-Pérez, J.I.	3	32	10.67	Universidad de Valladolid	Spain	2013	2017	3
Brian, A.	3	1	0.33	University of South Carolina	United States	2018	2018	3

AU: author; A: total number of articles; TC: total number of citations; TC/A: number of citations by article; C: country; 1st A: First article; Last A: Last article; H index: Hirsch index; (*) in this research topic.

**Table 6:** Most productive institutions in number of articles on the impact of the university classroom on managing the socio-educational well-being (2004–2018).

Institution	C	A	TC	TC/A	H Index *	1st A *	Last A *
Iowa State University	United States	12	137	11.42	6	2007	2018
The University of Hong Kong	Hong Kong	12	124	10.33	7	2005	2018
University of Texas at Austin	United States	12	334	27.83	8	2004	2018
University of Virginia	United States	12	466	38.83	7	2005	2017
University of Queensland	Australia	12	207	17.25	7	2004	2018
Ohio State University	United States	11	90	8.18	7	2006	2018
Brigham Young University	United States	11	400	36.36	7	2004	2017
University of Florida	United States	11	166	15.09	5	2007	2016
The University of Georgia	United States	11	182	16.55	7	2004	2018
Nanyang Technological University	Singapore	10	133	13.30	7	2009	2014

C: country; A: number of articles; TC: number of citations for all articles; TC/A: number of citations by article; H index: Hirsch index; 1st A: First article; Last A: Last article; (*) in this research topic.

**Table 7:** Most productive countries in number of articles on the impact of the university classroom on managing the socio-educational well-being (2004–2018).

Country	A	TC	TC/A	H Index	A				
					2004–2006	2007–2009	2010–2012	2013–2015	2016–2018
United States	787	9496	12.07	52	64	122	163	208	230
United Kingdom	154	1973	12.81	27	15	19	33	42	45
Australia	149	1460	9.80	21	15	22	32	38	42
Spain	92	608	6.61	15	1	6	15	32	38
Turkey	81	612	7.56	16	2	16	13	21	29
Canada	80	769	9.61	15	6	13	19	23	19
China	76	214	2.82	8	4	6	13	9	44
South Africa	46	181	3.93	7	1	4	6	16	19
Taiwan	43	415	9.65	12	0	10	9	11	13
Germany	38	406	10.68	13	1	6	6	11	14

A: number of articles; TC: number of citations for all articles; TC/A: number of citations by article; H index: Hirsch index in this research topic.

**Table 8:** Most productive countries and international collaboration (2004–2018).

Country	NC	Main Collaborators
United States	46	Canada, Australia, China, Taiwan, New Zealand
United Kingdom	32	Australia, Spain, Belgium, Greece, Netherlands
Australia	32	United States, United Kingdom, China, New Zealand, Japan
Spain	28	Poland, United States, Netherlands, United Kingdom, France
Turkey	26	United States, United Kingdom, China, New Zealand, Japan
Canada	9	United States, Australia, Hong Kong, United Kingdom, Japan
China	8	United States, Australia, United Kingdom, Chile, Cyprus
South Africa	7	United States, Australia, Belgium, Denmark, Georgia
Taiwan	4	United States, Australia, Singapore, United Kingdom
Germany	4	United States, Australia, China, Singapore

NC: number of collaborators.

**Table 9:** Main keywords on the impact of the university classroom on managing the socio-educational well-being (2004–2018).

Keyword	2004–2018		2004–2006		2007–2009		2010–2012		2013–2015		2016–2018	
	A	%	A	%	A	%	A	%	A	%	A	%
Human	437	22.0	28	20.7	67	24.5	82	22.3	122	24.0	138	19.8
Teaching	346	17.5	24	17.8	50	18.3	70	19.1	84	16.5	118	16.9
Education	332	16.8	22	16.3	53	19.4	49	13.4	87	17.1	121	17.3
Students	231	11.7	18	13.3	32	11.7	47	12.8	60	11.8	74	10.6
Organization and Management	213	10.7	12	8.9	43	15.8	43	11.7	53	10.4	62	8.9
University	211	10.6	18	13.3	24	8.8	41	11.2	74	14.5	61	8.7
Curriculum	171	8.6	11	8.1	26	9.5	30	8.2	54	10.6	49	7.0
Higher Education	154	7.8	1	0.7	11	4.0	29	7.9	43	8.4	70	10.0
Well-being	148	7.5	4	3.0	25	9.2	27	7.4	41	8.1	51	7.3
Learning	131	6.6	5	3.7	12	4.4	28	7.6	26	5.1	60	8.6
Faculty	122	6.2	6	4.4	15	5.5	32	8.7	54	10.6	15	2.1
Nursing Education	102	5.1	8	5.9	30	11.0	18	4.9	20	3.9	26	3.7
Classroom	95	4.8	8	5.9	17	6.2	11	3.0	21	4.1	38	5.4
Methodology	92	4.6	4	3.0	23	8.4	35	9.5	23	4.5	6	0.9
Integration	90	4.5	7	5.2	12	4.4	20	5.4	28	5.5	23	3.3
Psychology	88	4.4	0	0.0	2	0.7	4	1.1	29	5.7	51	7.3
Procedures	82	4.1	0	0.0	0	0.0	1	0.3	32	6.3	49	7.0
Program Evaluation	78	3.9	8	5.9	14	5.1	16	4.4	17	3.3	24	3.4
E-learning	75	3.8	3	2.2	9	3.3	18	4.9	13	2.6	31	4.4
Classroom Management	73	3.7	4	3.0	5	1.8	15	4.1	21	4.1	28	4.0

A: total number of articles; %: percentage of articles in which it appears.
